# Insulin Degrading Enzyme Induces a Conformational Change in Varicella-Zoster Virus gE, and Enhances Virus Infectivity and Stability

**DOI:** 10.1371/journal.pone.0011327

**Published:** 2010-06-25

**Authors:** Qingxue Li, Mir A. Ali, Kening Wang, Dean Sayre, Frederick G. Hamel, Elizabeth R. Fischer, Robert G. Bennett, Jeffrey I. Cohen

**Affiliations:** 1 Laboratory of Clinical Infectious Diseases, National Institutes of Health, Bethesda, Maryland, United States of America; 2 Research Service, Omaha VA Medical Center and the Department of Internal Medicine, University of Nebraska Medical Center, Omaha, Nebraska, United States of America; 3 Research Technology Branch, Rocky Mountain Laboratories, National Institutes of Health, Hamilton, Montana, United States of America; University of Cambridge, United Kingdom

## Abstract

Varicella-zoster virus (VZV) glycoprotein E (gE) is essential for virus infectivity and binds to a cellular receptor, insulin-degrading enzyme (IDE), through its unique amino terminal extracellular domain. Previous work has shown IDE plays an important role in VZV infection and virus cell-to-cell spread, which is the sole route for VZV spread in vitro. Here we report that a recombinant soluble IDE (rIDE) enhances VZV infectivity at an early step of infection associated with an increase in virus internalization, and increases cell-to-cell spread. VZV mutants lacking the IDE binding domain of gE were impaired for syncytia formation and membrane fusion. Pre-treatment of cell-free VZV with rIDE markedly enhanced the stability of the virus over a range of conditions. rIDE interacted with gE to elicit a conformational change in gE and rendered it more susceptible to proteolysis. Co-incubation of rIDE with gE modified the size of gE. We propose that the conformational change in gE elicited by IDE enhances infectivity and stability of the virus and leads to increased fusogenicity during VZV infection. The ability of rIDE to enhance infectivity of cell-free VZV over a wide range of incubation times and temperatures suggests that rIDE may be useful for increasing the stability of varicella or zoster vaccines.

## Introduction

Varicella-zoster virus (VZV), a member of the alpha-herpesvirus family, is the etiologic agent of chickenpox and shingles. In humans, cell-free virions are released from skin lesions and are transmitted to epithelial cells in the respiratory tract of susceptible hosts [1]. In cell culture, however, no cell-free infectious virions are spontaneously released, and infection is exclusively by cell-to-cell spread of virus. While cell-free virus can be obtained by sonication of infected cells, the lack of high titer cell-free virus has hindered the progress of studies to define the mechanism by which VZV enters into target cells.

Previous studies have identified cellular molecules that are important for entry of VZV into cells. Cation-independent mannose 6-phosphate receptor (MPR^ci^) has been proposed to facilitate an early step of VZV infection [2]. Previous studies from our laboratory showed that insulin-degrading enzyme (IDE), a member of the zinc metalloproteinase family, is a putative cellular receptor for VZV [3]. Down-regulation of IDE by specific siRNA, inhibition of IDE activity with bacitracin, or blocking IDE with antibody inhibited VZV infection and impaired cell-to-cell spread of the virus. Over-expression of human IDE by transfection into cell lines resulted in increased entry of both cell-free and cell-associated virus. VZV glycoprotein E (gE), which is essential for virus infectivity [4], [5], interacts with IDE through a binding domain located at the amino terminus of the ectodomain of gE that is not conserved in other human herpesviruses [3], [6], [7], [8]. VZV deleted for the IDE binding domain in gE is impaired for infectivity of cell-free virus [5] and shows reduced cell-to-cell spread of virus both in vitro and in human skin xenografts in SCID mice [5], [8]. Here, we show that the interaction of IDE with gE is important for VZV-induced syncytia formation and fusogenicity, and that recombinant soluble IDE (rIDE) modifies gE, induces a conformation change in gE, enhances VZV infectivity, and stabilizes cell-free virus.

## Results

### rIDE augments cell-free VZV infectivity at an early stage of infection and enhances stability of cell-free virus

**Figure 1:**
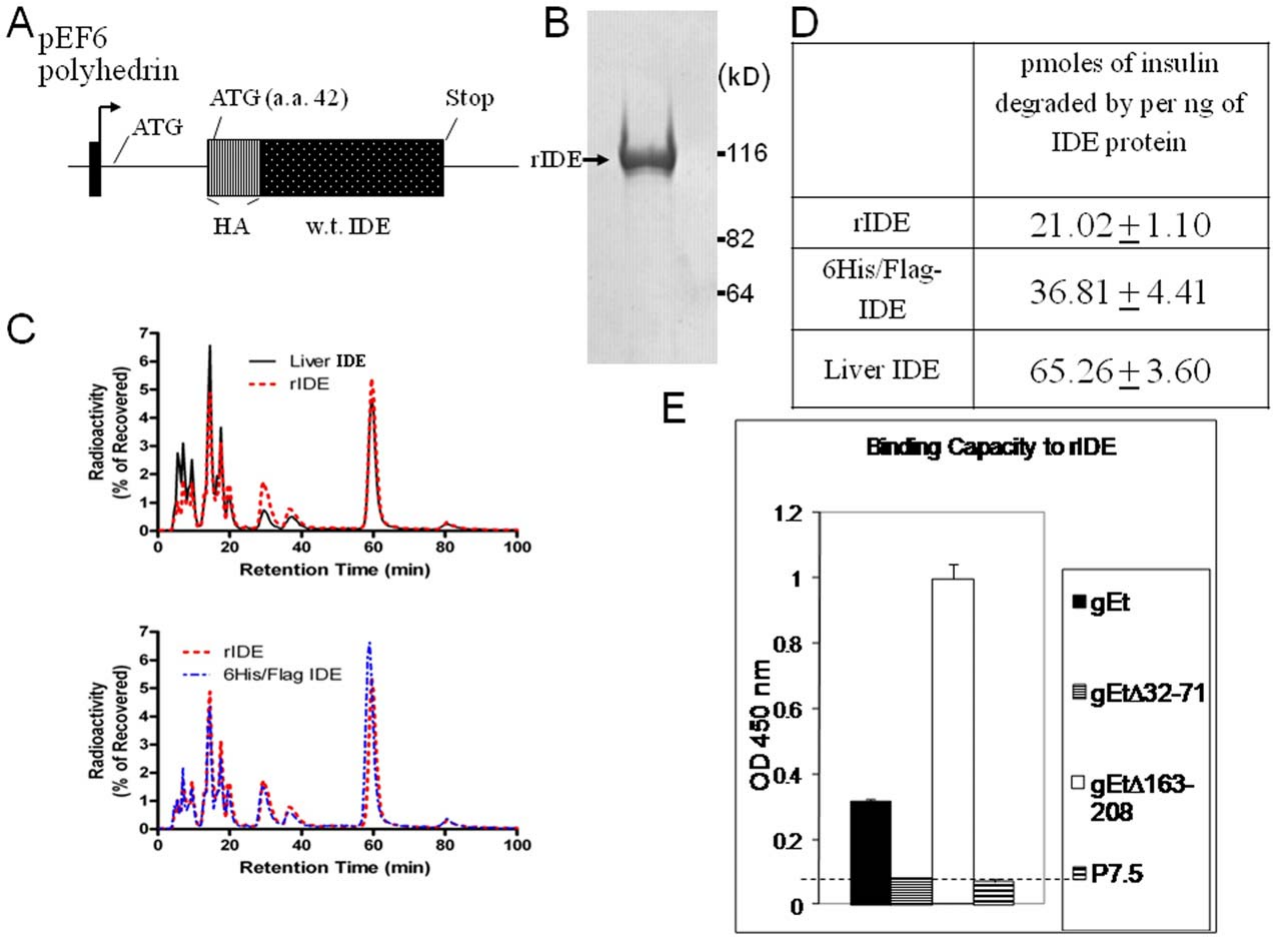
rIDE is expressed in insect cells, degrades insulin, and binds to VZV gE. (A) Structure of HA-tagged rIDE and its promoter in recombinant baculovirus. (B) rIDE was purified, eluted under native conditions, and stained with Coomassie Blue. Soluble rIDE is expressed as a 110 kDa protein in insect cells. (C) HPLC profiles of insulin degradation fragments observed after co-incubation of radiolabeled insulin with rIDE expressed in insect cells, endogenous IDE extracted from rat liver (top panel), or recombinant IDE (6His/Flag-IDE) expressed in eukaryotic cells (bottom panel). (D) Insulin-degrading enzymatic activities of IDE proteins. (E) ELISA assay for binding of rIDE to gEt, gEt lacking the IDE binding domain (gEtΔ32-71), gEt defective in binding to VZV gI (gEtΔ163-208), and a negative control protein (P7.5, vaccinia 7.5 protein). The horizontal dotted line indicates the level of nonspecific binding based on the control protein.

The open reading frame of human IDE contains two ATGs near the amino terminus that could serve as translation initiation codons. Previous studies with cloned IDE cDNA showed that the second ATG encoding amino acid 42, which better matches a Kozak consensus sequence, serves as the canonical start site for translation [9], [10]. Recombinant baculovirus was constructed to express human IDE with a hemaglutinin (HA) tag inserted after the second methionine (amino acid 42) of IDE driven by polyhedrin promoter [3], [11] (Fig. 1A). rIDE was expressed as a 110 kD protein (Fig. 1B), although gel filtration showed oligomerization of the protein as has been reported previously [12]. Incubation of rIDE with radiolabeled insulin resulted in a similar profile of degradation products as seen with endogenous IDE from rat liver [13] or another form of recombinant IDE [14] (Fig. 1C). rIDE had insulin degrading activity similar to recombinant 6HisFlag-IDE (Fig. 1D).

Previously we showed that IDE interacts with the extracellular domain of VZV gE, that a gE mutant lacking amino acids 32–71 cannot bind IDE, and that a gE mutant lacking amino acids 163–208 is unable to bind to VZV gI and shows enhanced binding to IDE [6]. Here we found that rIDE also formed a complex with the extracellular domain of gE, that rIDE did not interact with gE lacking the IDE binding domain, and that rIDE interacted to a greater extent with the mutant gE that does not bind gI (Fig. 1E).

**Figure 2:**
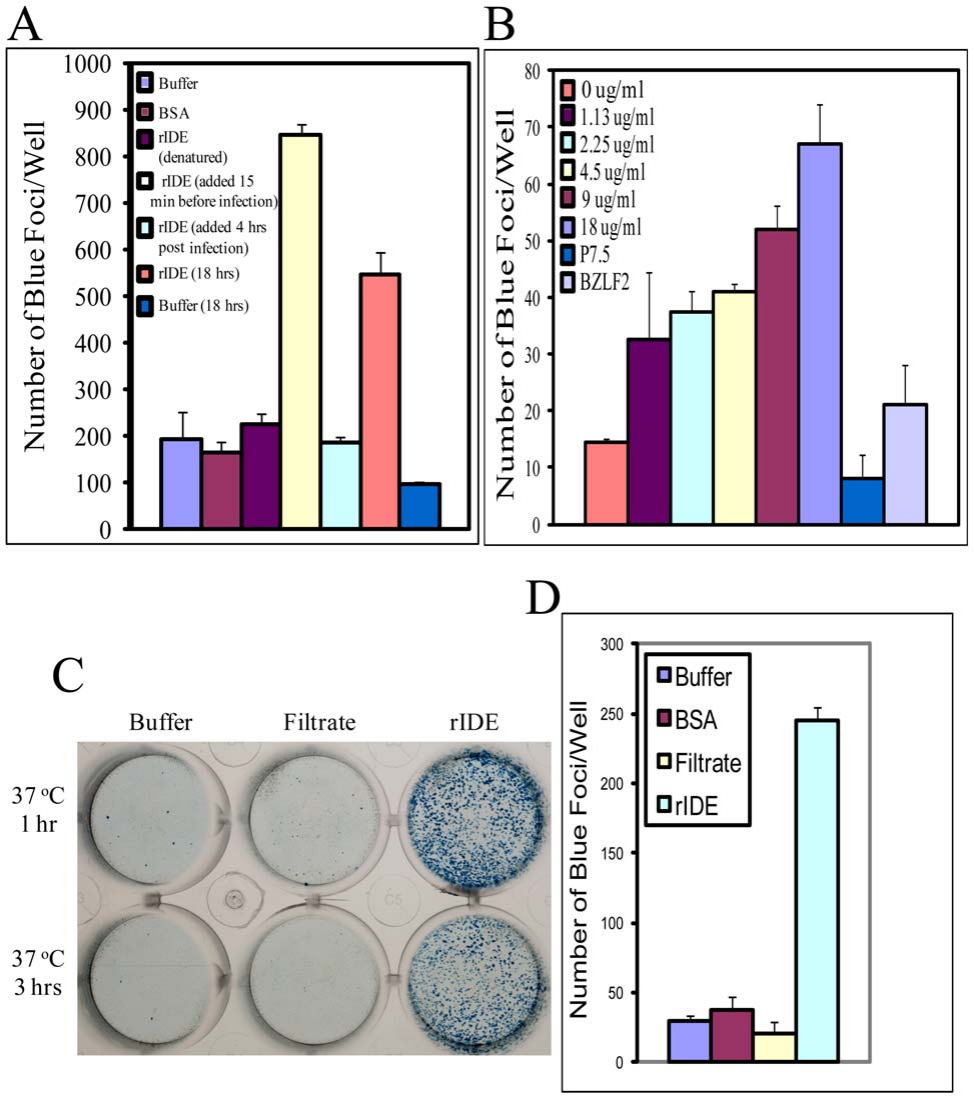
rIDE enhances VZV infectivity at an early stage of infection and increases the stability of virus. (A) Cell-free VZV (ROka-LacZ) virus was pre-treated with buffer, bovine serum albumin (BSA), rIDE (15 µg/ml), or denatured rIDE (IDE treated with acid and then base to a final neutral pH) at 37°C for 15 min before infecting melanoma cells. Infected cells were stained with X-gal at 3 days after infection to identify the number of VZV-positive foci. The experiment was repeated 10 times and a representative experiment is shown. For the 5^th^ bar, cells were infected with the same amount of virus as in bars 1-4, and rIDE was then added to the cells 4 hours post-infection. For the last two bars, the virus was incubated with rIDE or buffer at 37°C for 15 min and kept at 25°C for 18 hrs before infecting MeWo cells. (B) Cell-free ROka-LacZ was pre-incubated with increasing concentrations of rIDE (shown in µg/ml) at 37°C for 15 min and infected cells were stained with X-gal. The experiment was performed twice and one result is shown. The control proteins, P7.5 and BZLF2, were used at a concentration of 20 µg/ml. (C) Cell-free VZV (ROka-LacZ) was pre-incubated with rIDE, buffer, or filtrate passing through the rIDE purification column under the indicated conditions and infected cells were stained with X-gal at 4 days post-infection. The filtrate (control) consists of HA peptide which was used to elute rIDE from the purification column. (D) Cell-free ROka-LacZ virus was incubated with buffer, bovine serum albumen (BSA), filtrate that passed through without binding to a rIDE purification column, or rIDE for 18 hrs at 25°C before infecting cells and staining with X-gal. The experiment was performed 3 times, and a representative result is shown.

Previously, we reported that purified endogenous IDE protein extracted from liver blocked VZV infection, while rIDE from cloned cDNA expressed in baculovirus-infected cells enhanced VZV infectivity [3]. The difference in the activity of these two proteins could be due to the difference in their amino termini. rIDE begins at a canonical initiation codon at amino acid 42. Purified rIDE showed a single band of 110 kD while native IDE purified from liver is actually a complex mixture of IDE forms. To further investigate the effect of rIDE on VZV infectivity, we incubated cell-free VZV ROka-lacZ with rIDE or control proteins for 15 min at 37°C before infecting human melanoma cells with the virus. Three days later, the cells were fixed and treated with X-gal and blue foci were counted. rIDE significantly enhanced VZV infectivity compared with control protein or SPGC buffer alone (P<0.001 for rIDE vs. control protein, Student t test, Fig. 2A). Enhancement of infectivity was not observed when rIDE was added at 4 hr post-infection which bypasses the entry step (P<0.0001 for rIDE added at 15 min before infection vs. rIDE added 4 hr after infection by Student t test, Fig. 2A). Denaturation of rIDE with 12.5 mM citric acid at pH 2.3 followed by neutralization with 0.5 M HEPES buffer at pH 9.0 to a final pH 7.0, inactivated the enhancing activity of IDE. These results indicate that the native form of rIDE is required to enhance infectivity and that rIDE functions at an early stage of infection. Incubation of rIDE with cell-free VZV at 37°C for 15 min before adding the virus to cells enhanced infectivity in a dose-dependent manner (P = 0.0002 by ANOVA) (Fig. 2B).

**Figure 3:**
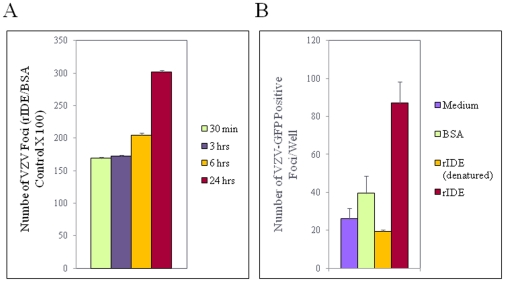
rIDE enhances VZV infectivity and increases the stability of virus. (A) Cell-free ROka-LacZ was pre-incubated with rIDE or BSA at 25°C for the indicated time before infecting MeWo cells. The ratio of the number of blue foci in cells infected with virus treated with rIDE or BSA(control) was determined. The experiment was preformed 11 times and a representative experiment is shown. Vertical lines indicate standard deviations. (B) Pre-treatment of VZV with rIDE at 4°C enhances viral infectivity. Cell-free ROka-GFP virus was incubated with medium, BSA, rIDE or denatured rIDE (rIDE treated with acid followed by base to a final neutral pH) for 60 min at 4°C before infecting cells. (P = 0.001 for rIDE vs. denatured rIDE, Student t test). Vertical lines indicate standard deviations. The experiment was performed 3 times, and a representative result is shown.

Since VZV is highly cell-associated in vitro, and cell-free virus produced by sonication is very labile and its infectivity declines quickly over time, we tested whether incubation of cell-free VZV with rIDE enhances stability of the virus. rIDE increased virus infectivity when incubated with cell-free ROka-lacZ for 1 or 3 hr at 37°C compared with buffer or with filtrate passing through the rIDE purification (Fig. 2C). rIDE also enhanced cell-free VZV infectivity and/or stability after incubation for 18 hrs at 25°C prior to infecting cells (P = 0.002 for rIDE vs. buffer or controls, Student t test) (Fig. 2A last two columns and Fig. 2D). Enhancement of virus stability by incubating rIDE with VZV was observed over a range of incubation periods at room temperature (Fig. 3A, P = 0.0002 for rIDE vs. BSA control for combined data from 11 experiments (each with ≥2 replicates). Incubation of rIDE and cell-free virus at 4°C for 60 min before infection of cells also augmented VZV infectivity (Fig. 3B). These results indicate that rIDE enhances both infectivity and stability of cell-free VZV over a wide range of incubation times and incubation temperatures.

**Figure 4:**
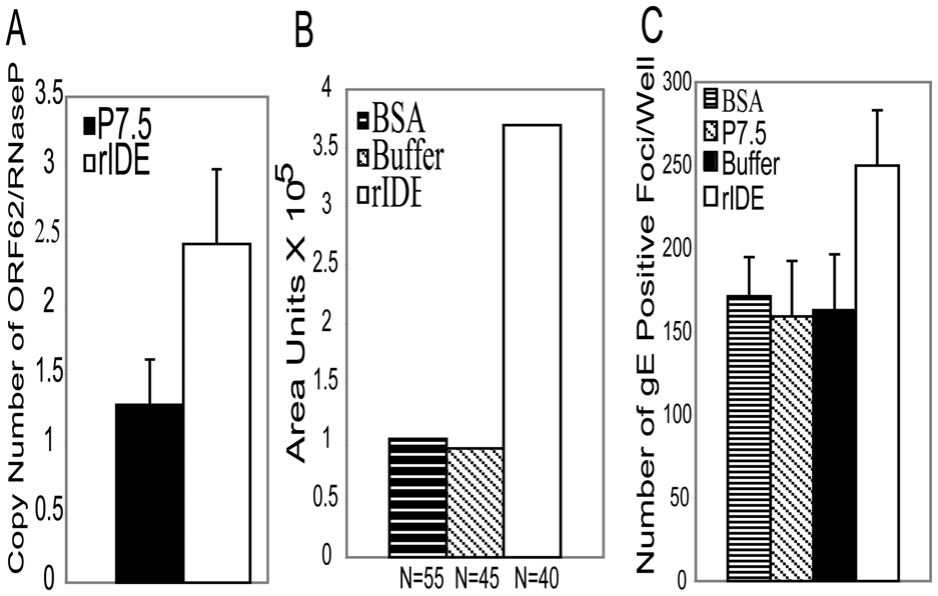
rIDE promotes VZV internalization during entry and cell-to-cell spread of virus. (A) Cell free ROka-lacZ was incubated with P7.5 control protein or rIDE for 30 min at 37°C, the mixture was incubated with cells for 60 min on ice to allow binding followed by 90 min at 37°C for entry, and DNA was then extracted from the cells and the copy number of intracellular VZV genomes was determined by real-time PCR. The ratio of VZV ORF 62 DNA to cellular RNaseP DNA is shown on the y axis. The experiment was performed three times and a representative result is shown. (B) Melanoma cells were infected with ROka-lacZ, 2.5 hrs later the inoculum was removed by washing with PBS, rIDE was added to the cells, and 3 days later the cells were stained with X-gal. Blue VZV-infected foci were photographed at a magnification 40X and plaque sizes were measured with ImageJ software as previously described [3]. N is the number of plaques measured. (C) Cell-free VZV from a commercial lot of zoster vaccine was incubated with rIDE for 30 min, melanoma cells were infected with the mixture for 4 days, and the cells were fixed and stained with anti-gE monoclonal antibody and infected foci were visualized by fluorescence microscopy. The graph represents pooled data from three independent experiments. Vertical lines indicate standard deviations.

To further determine if the effect of rIDE on VZV infectivity is at an early step of virus infection, we performed quantitative PCR for VZV DNA to determine the copy number of viral genomes inside the cells 90 min after infection. Incubation of rIDE with virus prior to infection resulted in increased numbers of VZV genomes inside the cells, compared with virus incubated with a control protein, indicating that rIDE increases virus internalization (P<0.0001 for rIDE vs. P7.5, Fig. 4A). Addition of rIDE at 2.5 hr after infection with VZV resulted in an increase in the size of plaques (P<0.0001 for both rIDE vs. BSA control and rIDE vs. Buffer, Fig. 4B), suggesting that rIDE not only augments infectivity of cell-free virus at entry but also promotes infectivity through enhanced cell-to-cell spread of virus. This is consistent with our earlier observation that IDE is important for infection with both cell-free and cell-associated VZV [3].

Since the prior experiments were all performed using recombinant-derived VZV, we also tested the effect of rIDE on non-recombinant viruses. Cell-free zoster vaccine virus, which contains various stabilizers, was reconstituted according to the manufacturer's instruction, and incubated with rIDE or control proteins for 30 min at 37°C. rIDE increased the infectivity of zoster vaccine virus (P<0.0001 for rIDE vs. buffer, bovine serum albumen (BSA), or P7.5 control protein by Student t test; Fig. 4C).

### rIDE modifies VZV gE and induces a conformational change in gE

**Figure 5:**
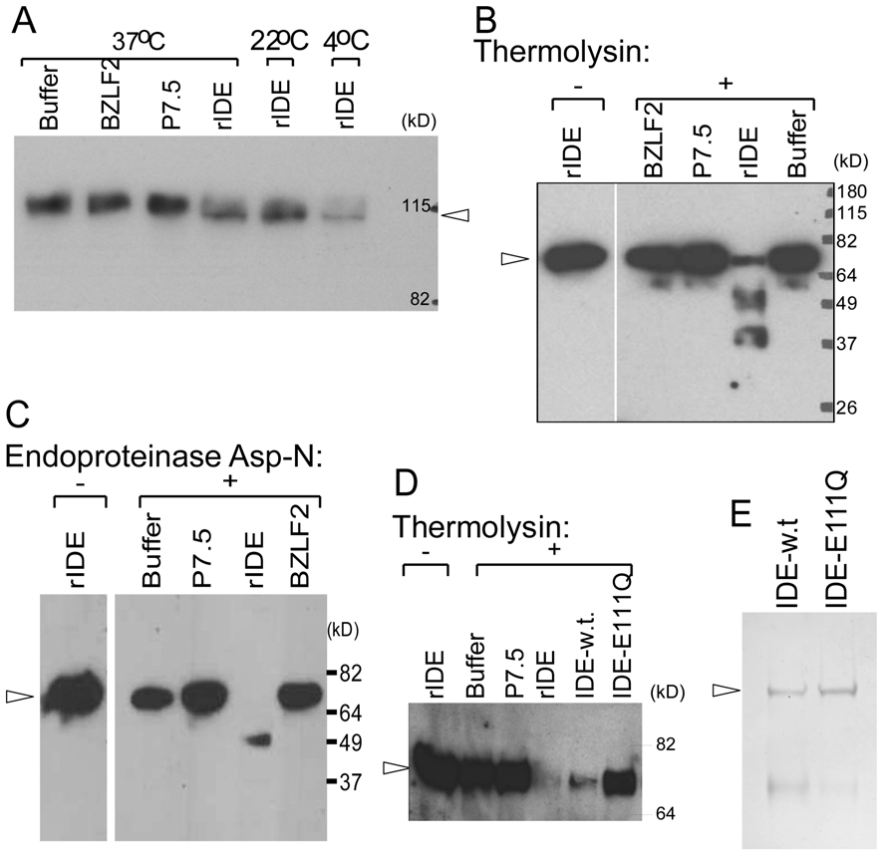
rIDE modifies gEt and induces a conformational change in gEt. (A) Biotin labeled gEt-Fc protein (arrow) was incubated with rIDE at the indicated temperature for 30 min. After electrophoresis and transfer onto a nitrocellulose membrane, proteins were visualized with streptavidin-conjugated-horse radish peroxidase. (B) Biotin-labeled gEt-His protein (arrow) was incubated with buffer, control protein BZLF2 or P7.5, rIDE (produced in baculovirus) at 37°C for 30 min, followed by urea for 18 hr and thermolysin for 30 sec. The proteins were separated by electropheresis, transferred to a nitrocellulose membrane and proteins were detected using streptavidin-conjugated horse radish peroxidase. (C) Biotin-labeled gEt-His protein (arrow) was incubated with rIDE or control proteins at 37°C for 30 min, followed by incubation with 4 µg/ml of endoproteinase Asp-N (Roche Applied Science, Indianapolis, IN) at 37°C for 45 sec. The digestion was then terminated by adding 0.5 M EDTA and samples were boiled in SDS-PAGE gel loading buffer with 2.5% 2-mercaptoethanol and separated by electrophoresis. Protein fragments were detected by streptavidin conjugated-horse radish peroxidase. (D) Binding of catalytically inactive IDE mutant protein IDE-E111Q to gEt fails to induce a conformational change in gE. Biotin-labeled gEt-His protein (arrow) was incubated with rIDE (produced by baculovirus), IDE-E111Q (produced in bacteria), or IDE-w. t. (produced in bacteria), or negative control proteins as indicated at 37°C for 30 min, followed by thermolysin and processed as described in panel B. (E) Coomassie Blue stained SDS-PAGE gel showing the amount of IDE-E111Q and IDE-w.t. proteins used for pulse proteolysis assay in panel D.

IDE is a metalloproteinase. While IDE interacts with a variety of substrates, it cleaves only a subset of its substrates such as insulin and β-amyloid protein at perceptible rates [15], [16]. Previously, we incubated the extracellular domain of gE (gEt) with IDE protein extracted from liver and did not detect cleavage or degradation of gEt using a monoclonal antibody [3]. To determine if rIDE modifies gE, we incubated biotin-labeled gEt protein with rIDE or control proteins (P7.5-Fc which encodes vaccinia virus P7.5 protein or BZLF2-Fc which encodes Epstein-Barr virus BZLF2 protein) at 37°C, 22°C, or 4°C for 30 min. The proteins were then boiled in sample buffer, separated on an SDS-PAGE gel, and gEt was stained with streptavidin conjugated-horse radish peroxidase. Incubation of gEt with rIDE, but not with the control proteins, resulted in a slightly smaller sized band than full length gEt, indicating that rIDE modifies gEt (Fig. 5A). While it was somewhat surprising that rIDE modified gE after incubation at 4°C, this is consistent with our observations that rIDE enhanced VZV infectivity at 4°C (Fig. 3B).

Many viral glycoproteins undergo conformational changes after binding their receptor or co-receptor which results in the exposure of fusion peptides embedded in the viral glycoproteins that initiate a series of events leading to fusion between the viral envelope and the cell membrane [17], [18]. Since VZV gE has an important role in syncytia formation and membrane fusion [19], we postulated that gE might undergo a conformational change after binding to rIDE. Limited exposure of ligand-receptor complexes to proteinase has been used to detect receptor-induced conformational changes in several proteins, including avian sarcoma/leucosis virus (ALV) envelope protein complexed with its receptor Tva and spike protein of mouse hepatitis virus complexed with soluble receptor, CEACAM1a [20], [21], [22]. The extracellular domain of gE was labeled with biotin and incubated with rIDE, buffer, or control protein (P7.5-Fc) at room temperature for 30 min followed by addition of thermolysin, a proteinase that hydrolyzes peptide bonds at hydrophobic residues, for 30 sec. Proteins were separated on an SDS-PAGE gel and incubated with streptavidin conjugated-horse radish peroxidase. Incubation of gEt with rIDE, but not with buffer or control protein, resulted in a reduction in the intensity of the 70 kDa gEt band (Fig. 5B and 5D). Similar results were also observed with endoproteinase Asp-N digestion (Fig. 5C). Therefore, rIDE triggered a conformational change in VZV gEt that confers an increased susceptibility to proteinase. Repeated attempts to determine a cleavage site on gE by N-terminal protein sequencing failed to generate any sequences.

**Figure 6:**
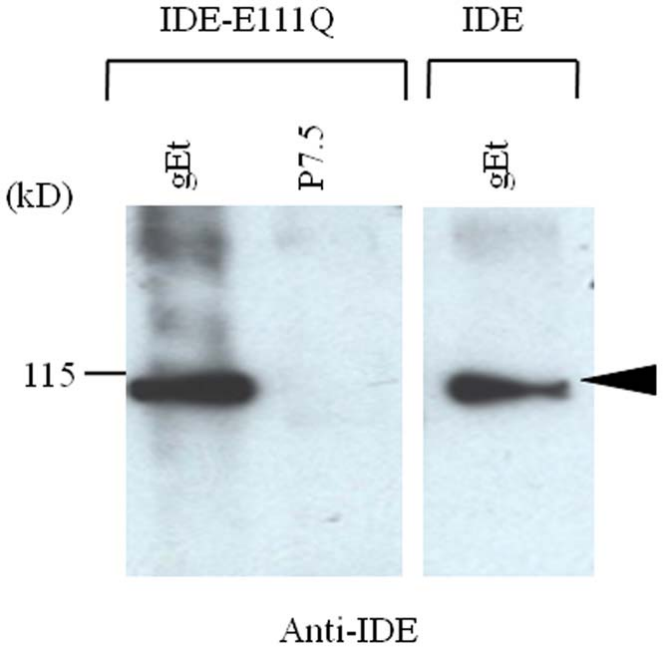
IDE mutant protein, IDE-E111Q binds to gEt. gEt but not control protein p7.5, pulled down both IDE-E111Q protein and wild-type IDE (arrow).

Glutamic acid 111 in the zinc-binding site of human IDE is a major catalytic residue for its insulin degrading function [23]. Mutation of glutamic acid 111 to glutamine in IDE results in a mutant protein, IDE-E111Q, which is catalytically inactive for insulin degradation. Although IDE-E111Q formed a complex with gEt (Fig. 6), it failed to elicit a conformational change in gEt as compared with rIDE or wild-type IDE in the thermolysin assay (Fig. 5D, E). These results suggest that the catalytic activity of IDE for insulin degradation might be important for inducing a conformational change in gE.

### A VZV mutant lacking the IDE binding domain of gE is impaired for membrane fusion and syncytia formation

**Figure 7:**
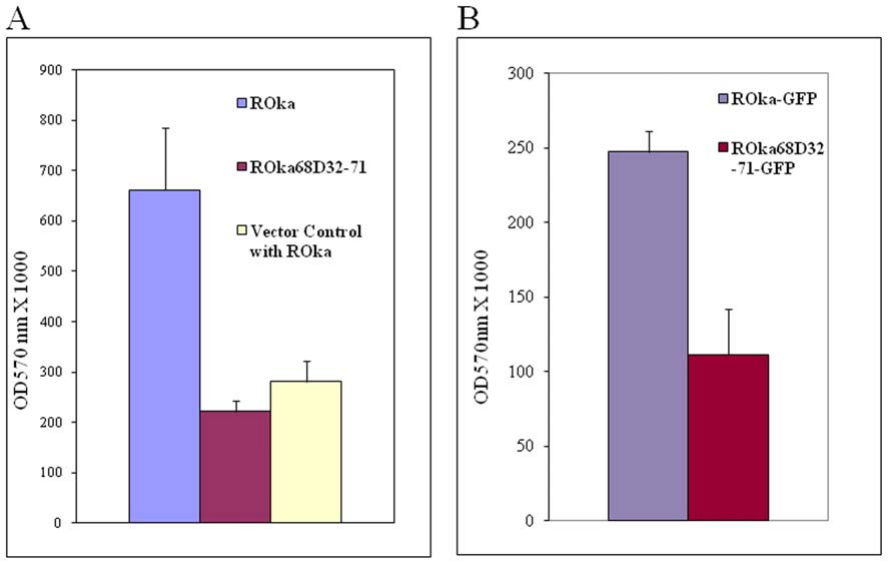
VZV mutant lacking the IDE binding domain of gE shows reduced fusogenicity by membrane fusion assay. (A) Melanoma cells expressing T7 polymerase were infected with VZV ROka, ROka68D32-71, and the cells were incubated with HeLa cells containing the β–galactosidase gene driven by the T7 promoter or a control GFP plasmid for 20 hr. The cells were lysed, incubated with chlorophenol red-β-D-galactopyranoside, and β–galactosidase activity was measured using a spectrophotometer at OD570 nm. The experiment was also performed at 16 hr and 24 hr and similar results were obtained. Vertical lines indicate standard deviations. (B) Human melanoma cells expressing T7 polymerase were infected with the same MOI of ROka-GFP or ROka68D32-71-GFP, and cells were co-incubated with HeLa cells containing the β–galactosidase gene driven by the T7 promoter for 16 hrs. The cells were lysed, incubated with chlorophenol red-β-D-galactopyranoside, and β–galactosidase activity was measured using a spectrophotometer at OD570 nm. The experiment was performed twice with similar results. (P<0.001 for ROka-GFP vs. ROka68D32-71-GFP, Student t test). Vertical lines indicate standard deviations.

To determine if the interaction of gE with IDE is important for membrane fusion, we used a reporter system in which VZV permissive HeLa cells were transfected with a plasmid encoding β-galactosidase under the T7 promoter (pG1N-T7-β-gal) and served as target cells. One day after transfection, the target cells were incubated for 20 hr with melanoma cells stably expressing T7 polymerase that contained equal titers (10^5^ PFU/ml) of control VZV (ROka) or a mutant virus, ROka68D32-71 that lacks the IDE binding domain (amino acids 32 to 71) of gE. Incubation of target cells with ROka68D32-71-infected cells resulted in reduced membrane fusion activity as measured by lower levels of β-galactosidase compared with ROka-infected cells (P = 0.0001 for ROka vs. ROka68D32-71 by Student t test; Fig. 7A). Similar results were observed with ROka68D32-71-GFP (which also expresses GFP) and ROka-GFP (Fig. 7B). Transfection of HeLa cells with vector control plasmid, in place of the reporter plasmid, followed by infection with VZV ROka resulted in a background level of fusion activity. Therefore, VZV mutants lacking the IDE binding domain of gE have reduced membrane fusogenicity.

**Figure 8:**
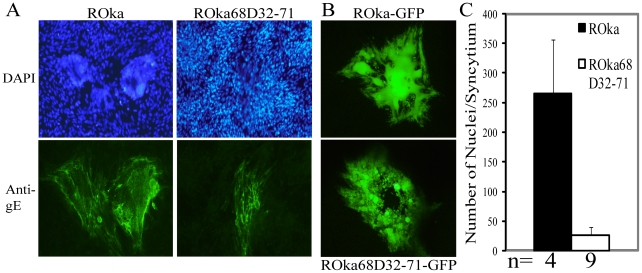
VZV mutants deleted for the IDE binding domain of gE are impaired for syncytia formation. (A) Melanoma cells were infected with VZV ROka or ROka68D32-71 and stained with DAPI (top panels) or mouse monoclonal antibody to gE followed by anti-mouse-Alexa-488 and visualized by immunofluorescence microscopy. Magnification 40X. (B) Melanoma cells were infected with VZV ROka-GFP or ROka68D32-71-GFP and visualized directly by fluorescence microscopy. Magnification 40X. (C) Quantification of the number of nuclei in syncytia per nucleus was performed using confocal microscopy (Leica SP2, Leica Microsystems, Exton, PA). Sequential Z-sections of DAPI stained infected cells were acquired for 3D reconstruction of representative cells with Imaris software (version 6.2, Bitplane AG, Zurich, Switzerland). The numbers of nuclei were automatically determined using spot function in Imaris software and manually corrected for errors by independent investigators in Biological Imaging, Research Technologies Branch, NIH. Vertical lines show standard deviations. n represents the number of syncytia analyzed in which nuclei were counted by a microscopist who was unaware of the expected outcome of the experiment.

To further examine the function of IDE in VZV infection, we analyzed syncyctia formation in cells infected with ROka68D32-71 and ROka68D32-71-GFP. While the truncated gE in ROka68D32-71 virus is impaired for binding to IDE in a pull-down assay, the mutant virus is not impaired for maturation and egress to the cell surface [5]. Both ROka68D32-71 and ROka68D32-71-GFP showed smaller syncytia with fewer nuclei in the syncytia compared with their parental viruses, ROka or ROka-GFP (Fig. 8A and B). Cells infected with ROka68D32-71-GFP had significantly fewer nuclei per syncytium than cells infected with ROka-GFP when the total number of nuclei in syncytia were quantified using 3 dimensional reconstruction of sequential Z-sections of cells with confocal microscopy (Fig. 8C, p<0.001).

### A VZV mutant lacking the IDE binding domain of gE accumulates on the cell surface or the cell-cell junction

**Figure 9:**
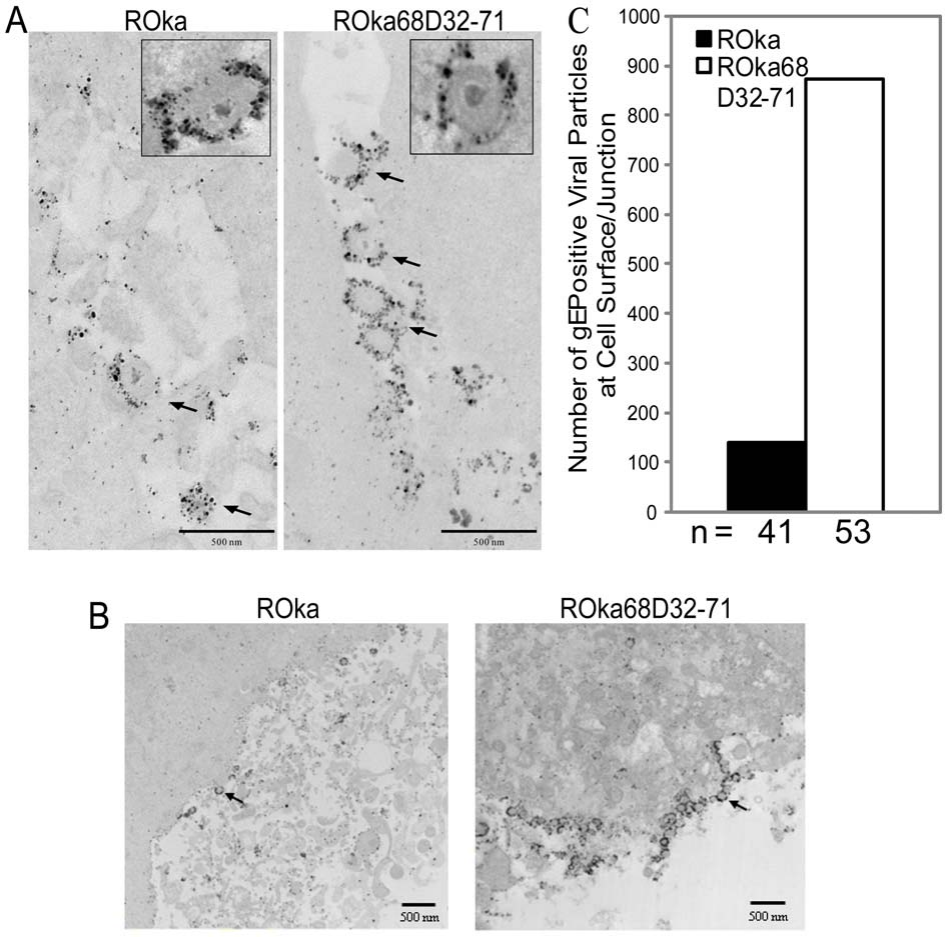
Accumulation of a VZV mutant deleted for the IDE binding domain of gE at the cell surface and at the cell-cell junction. Melanoma cells were infected with VZV ROka or ROka68D32-71, fixed, and incubated with monoclonal antibody to VZV gE followed by FluoroNanogold-conjugated anti-mouse antibody and visualized by transmission electron microscopy. (A) Representative virions at cell-cell junctions are indicated with arrows. Magnification 12000×. The insets in panel A provide higher-power images of selected virions. (B) Representative virions on the cell surface are indicated with arrows. Magnification 5000×. (C) Quantification of gE-positive virions on the cell surface and at cell-cell junctions. Multiple cells from the experiment in panel A were coded and observed under a Hitachi H7500 transmission electron microscope. gE positive viral particles at cell surface and/or junctions were counted.

**Figure 10:**
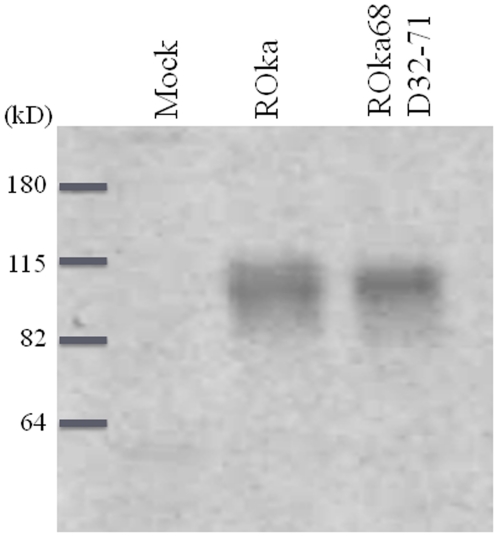
Deletion of IDE binding domain on gE (amino acids 32–71) does not affect myristylation of gE. ROka or ROka68D32-71 infected cells were radiolabeled with ^3^H-myristic acid and immunoprecipitated with anti-gE antibody.

To rule out the possibility that the reduced level of syncytia formation of the VZV mutant might be due to defective maturation and transport to cell surface and/or to cell-cell junctions, we performed immuno-electron microscopy studies. Melanoma cells infected with ROka68D32-71 showed large numbers of enveloped virions accumulating at cell-cell junctions (Fig. 9A) and on the cell surface (Fig. 9B). In 53 randomly selected immune-electron micrographs, there were a total of 873 gE-positive ROka68D32-71 virions on the cell surface and/or in cell junctions, compared with a total 140 gE-positive ROka virions in 41 pictures (Fig. 9C, P<0.00001 for ROka68D32-71 vs. ROka), indicating that the impairment in syncytia formation and cell-to-cell spread of the IDE binding domain mutant virus is not due to defective maturation with reduced transport to the cell surface or to cell-cell junctions. A previous study showed that VZV gE is myristylated [24]. Myristylation of proteins is important for their association with membranes. To rule out the possibility that deletion of the IDE binding domain of gE does not adversely affect gE myristylation, we labeled ROka or ROka68D32-71 infected cells with ^3^H-myristic acid and immunoprecipitated gE. gE from both parental and mutant VZV showed similar levels of myristylation, indicating that differences in myristylation do not explain the phenotype observed with the mutant virus (Fig. 10). Therefore, the IDE binding domain of gE is important for membrane fusogenicity, syncytia formation, and for cell-to-cell spread of the virus.

## Discussion

We have shown that soluble rIDE enhances VZV infectivity and cell-to-cell spread in human melanoma cells at an early step of virus infection. rIDE interacted with gE to elicit a conformational change in gE and modified the size of gE. In addition, a VZV mutant virus lacking the IDE binding domain of gE was impaired for syncytia formation and membrane fusion, suggesting that IDE enhances infectivity and stability of the virus through eliciting a conformational change in gE and modulating fusogenicity during VZV infection.

Previous studies using fibroblasts and melanoma cells suggested that IDE functions as a cellular receptor for gE enhancing VZV infectivity and cell-to-cell spread in these two cell types [3], [5]. However, a recent study found that although a VZV mutant with a deletion in the IDE binding domain of gE showed reduced cell-to-cell spread of virus in melanoma cells and impaired infectivity of skin xenografts in vivo, it was not defective in infecting T cells [8], suggesting that IDE may facilitate virus entry in a cell-type dependent manner, similar to gp42 of Epstein-Barr virus [25]. For the closely related herpes simplex virus (HSV), cellular molecules have also been shown to preferentially mediate virus entry in different cell types; nectin-1 is important for entry of HSV in neurons, while HVEM is used for entry in lymphocytes [26].

VZV gE binds to IDE through its amino-terminal region, although the overall conformation of gE is important for its interaction with IDE [6], [8]. The observation that gE produced by the baculovirus expression system is smaller in size than its counterpart produced from mammalian cells (Li et al., unpublished data) suggests that it is less glycosylated in insect cells. gE produced by baculovirus forms a complex with IDE [3], which implies that glycosylation of gE may not be important for interaction with IDE. Carpenter et. al. reported that IDE binds to the mature 98-kDa form of VZV gE under low salt conditions, but IDE binds only to the 73 kDa form of gE at high salt conditions [27]. They showed that tunicamycin blocks glycosylation of gE in the endoplasmic reticulum resulting in only production of the 73 kDa form of gE, and that in the presence of tunicamycin, gE was blocked in its transit out of the endoplasmic reticulum. Therefore they concluded that IDE must interact only with the 73 kDa form of gE in the cytosol and that this binding does not represent a receptor/ligand interaction. Interestingly, a previous study by these authors showed that the smaller form of gE is present on cell surface, recycles through the endocytotic pathway, and is incorporated into virions [28]. Studies from other groups also showed that a mixture of forms of gE ranging from ∼50–100 kDa are present in purified virions [29], [30], [31]. Therefore, the 73 kDa form of gE is present in virions where it may interact with IDE.

**Figure 11:**
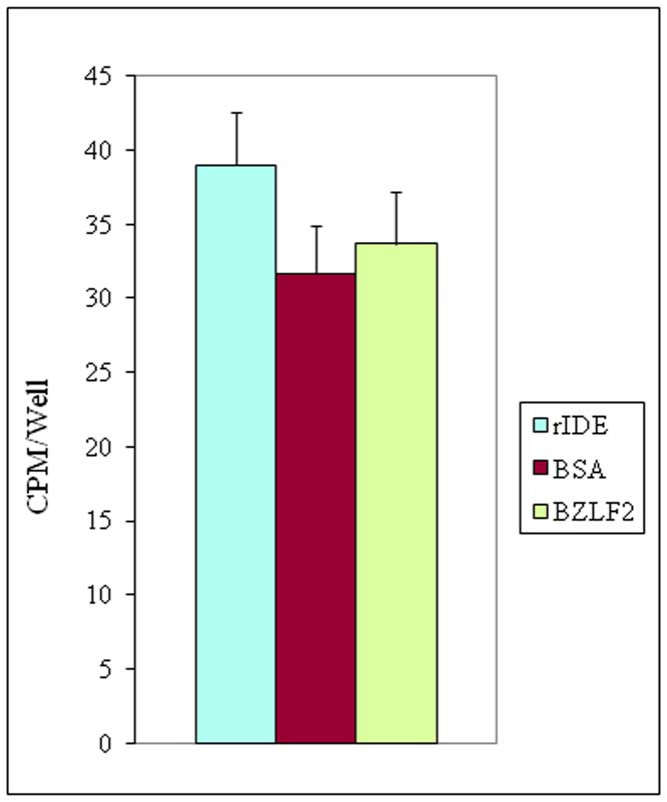
Treatment of VZV with rIDE does not increase virus binding. POka (parental Oka VZV) or mock infected MRC-5 cells were metabolically labeled with ^3^H-thymidine at 0.25 mCi/ml for 36 hr beginning at 8 hr post-infection, and cell-free virus was prepared by sonication as described above. The virus was then pre-incubated with rIDE or control proteins at 37°C for 30 min before addition to human melanoma cells on ice for 80 min in the presence of 100 µg/ml of heparin (to block binding through cell surface heparin sulfate). After extensive washing with cold PBS, the cells were lysed and radioactivity bound to cells was measured. The number of radioactive counts per minute for mock-infected MRC-5 cells was subtracted from the counts per minute for POka-infected cells (P = 0.1 for rIDE vs. BSA or for rIDE vs. BZLF2, Student t test). The data shown are based on two independent experiments.

Soluble receptor-mediated enhancement of virus infectivity has been reported for several viruses including HIV, avian leukosis/sarcoma virus, mouse coronavirus, [32], [33], [34], [35] and the closely related herpes simplex virus (HSV). Nectin-1 and 3-*O*-sulfated heparan sulfate function as receptors for HSV [36], [37]. Soluble 3-*O*-sulfated heparin sulfate triggers HSV entry into non-permissive cells and augments viral glycoprotein-mediated membrane fusion [38]. A soluble truncated form of nectin-1 lacking the transmembrane domain confers HSV susceptibility to non-permissive cells [39]. Further studies showed that the soluble V domain of nectin-1, (amino acid residues 1–123), promotes HSV entry. Interestingly, in this study neither free nor gD-bound sNec1_123_ was bound to the cell surface, suggesting that the interaction of soluble receptor with its gD ligand facilitates virus penetration by activating viral fusion machinery rather than directly enhancing virus attachment to cells [40]. While rIDE increased VZV infectivity at an early stage of infection, treatment of radiolabeled VZV with rIDE did not enhance virus binding (Fig. 11). Thus, soluble rIDE appears to promote VZV infectivity by increasing the efficiency of entry of virus into cells, rather than enhancing attachment. This conclusion is further supported by the observation that rIDE increases internalization of VZV DNA within 90 min of initiating infection.

We found that rIDE induces a conformational change in VZV gE. Numerous studies have established that receptor binding-induced conformational change in viral glycoproteins is critical in initiating virus-mediated membrane fusion. The conformational change results in exposed viral fusion peptides and provides energy for the fusion process through refolding [18], [41], [42]. For larger viruses such as HSV, the fusion machinery consists of more than one glycoprotein working in concert [43], [44]. Since VZV gE is not fusogenic when expressed alone, but does act as a fusogen in concert with other glycoproteins [45], it is possible that the conformational changes elicited by rIDE may either directly enhance fusogenicity of gE, or allow recruitment of glycoproteins gB and gH, to form a fusion complex.

In addition to the receptor-binding mediated conformational change of viral glycoprotein, sequential conformational changes induced by proteolysis are required during the entry process of several viruses. Proteases cleave viral fusion proteins, including glycoproteins, into mature, fusogenic forms and enhance virus binding and infectivity [46], [47], [48], [49], [50], [51], [52], [53]. Our finding that rIDE, a metalloproteinase, triggers a conformational change in gE and modifies the size of gE suggests that a proteolytic activity of IDE might be important for VZV infectivity. This is further supported by the fact that the IDE mutant (IDE-E111Q) which is impaired for degradation of insulin, fails to induce a conformational change in gE. Since gE is much larger than insulin and gE lacks any detectable amino acid homology with other substrates of IDE, the interacting amino acid residues of IDE with insulin and gE are likely to be different. Therefore, there may be a proteolytic motif of IDE for gE which is different from that for degrading insulin.

HSV enters cells through different pathways involving either the cell surface membrane or intracellular vesicles such as endosomes in a cell-type dependent manner [26], [54], [55]. Previous work by Hambleton and colleagues suggested VZV enters human fibroblasts through an endocytosis pathway [56]. Although we have shown that rIDE enhances VZV internalization, it is not clear whether this occurs at the cell surface or in endosomes. In most cell types, endogenous IDE localizes primarily in endosomes and other subcellular compartments with about 10% on the cell surface or in extracellular spaces. It is interesting to note that upon receptor binding, activation of coronavirus glycoprotein by extracellular proteases facilitates virus entry through the cell surface plasma membrane, while in the absence of extracellular proteases the virus enters the cell using the endosomal pathway [22].

We found that rIDE increased the stability of cell-free VZV. There are two possible explanations for how rIDE might enhance virus stability. First, interaction of a virus glycoprotein with its receptor may convert the glycoprotein from its native state to a new metastable state resulting in a long-lived envelope protein-receptor intermediate. ALV pre-loaded with a form of soluble receptor, Tva/Tvb, is very stable at 37°C [57]. Second, pre-treatment of virus with its receptor may target the virus to enter cells through a pathway that is more favorable for infection. ALV bound to the glycophosphatidylinositol-anchored form of its receptor (Tva) accumulates in a fusion compartment where it is more stable than if it enters though the transmembrane-anchored form of the receptor [58]. Although endogenous IDE is found both on the cell surface and in endosomes, it does not contain a predicted membrane anchoring domain. It has been postulated, however, that IDE might contain a glycophosphatidylinositol anchor [59].

Cell-free VZV is usually produced in vitro by sonicating infected cells and the virus is very labile. The current varicella and zoster vaccines used in the United States are supplied as lyophilized viruses that must be stored at −20°C or colder, and used within 30 minutes after they are reconstituted in aqueous solution [60]. rIDE might be useful as an additive for cell-free VZV vaccines to improve their stability.

We found that VZV lacking the IDE binding domain of gE is impaired for virus-induced membrane fusion, syncytia formation and cell-to-cell spread, even though gE-positive virions accumulate on the cell surface and at cell junctions. Transmission of VZV in vitro occurs exclusively by cell-to-cell spread. Grose and colleagues have postulated that VZV has evolved viral glycoproteins geared more towards cell-to-cell fusion to mediate syncytia formation rather than towards virus-to-cell fusion as seen in HSV [19]. Several studies suggest that gE has an important role in virus-induced membrane fusion during infection [19], [61]. gE promotes fusion when co-expressed with either gB or gH in transfected cells or using a vaccinia virus expression system [45], [62]. Berarducci et al. described a VZV mutant (rOka-P27-Y51) with a deletion in part of the IDE binding domain of gE that shows reduced cell-to-cell spread [7]. Our findings that a VZV mutant deleted for the IDE binding domain is impaired for syncytia formation and membrane fusion provides further evidence for the role of gE both as a receptor binding molecule and as a contributor to fusogenicity. We propose that the interaction of IDE with gE results in receptor binding and a conformational change that may include proteolysis. This process would lead to enhanced fusogenicity either alone or in conjunction with the recruitment of other viral glycoproteins during entry of VZV into target cells.

## Materials and Methods

### Cells and viruses

Human melanoma cells (MeWo [61], from Charles Grose, University of Iowa) and fibroblast cells (MRC-5, American Type Culture Collection, Manassas, VA) were grown in Minimum Essential Medium with 10% fetal bovine serum (FBS). HeLa cells (American Type Culture Collection) were maintained in Dulbecco's Modified Eagle Medium with 10% FBS. SF9 or H5 (*Spodoptera frugiperda*) insect cells were cultured in TNM-FH medium (BD Biosciences Pharmingen, San Diego, CA).

VZV strains ROka (recombinant derived Oka), ROka-lacZ (ROka expressing beta-galactosidase) [63], ROka-GFP [3], and VZV mutant ROka68D32-71 [5] were grown on MeWo cells. ROka68D32-71-GFP was constructed by inserting a cassette containing the human cytomegalovirus promoter driving green fluorescence protein (GFP) (from plasmid pEGFP-N1, Clontech-BD Biosciences, Palo Alto, CA) into the AvrII site of cosmid VZV MstIIA-68D32-71 located between VZV ORF65 and ORF66. The resulting cosmid was tranfected into melanoma cells along with plasmid pCMV62 and cosmids NotI A, Not IB, and Mst IIB, and the virus obtained was termed ROka68D32-71-GFP.

Cell-free VZV virus was prepared from VZV-infected MRC-5 cells that displayed 50–80% cytopathic effects (CPE) by scraping cells from flasks in SPGC buffer (10% FBS, 0.1% sodium glutamine, 5% sucrose in PBS), freeze-thawing the cells once, sonicating the lysate, centrifuging the lysate at 1,240×g for 10 min at 4°C, and transferring the supernatant to a new tube as virus stock. Cell-free zoster vaccine (Zostavax, Merck, Whitehouse Station, NJ) was reconstituted in sterile water following the manufacturer's instructions and used for some experiments.

### VZV infectivity assay

Cell-free VZV virus encoding β-galactosidase or GFP was incubated with rIDE or other proteins and added to MeWo cells in SPGC buffer at 33°C for 90 min before changing to normal growth medium. Infected foci were scored at day 3–5 post-infection by staining with 5-bromo-4-chloro-3-indolyl β-D-galactopyranoside (X-gal).

Cell-free vaccine virus was reconstituted in sterile water (as recommended by the manufacturer) and added to MeWo cells as described above. Four days after infection, the cells were fixed and stained with anti-gE monoclonal antibody (Chemicon, Temecula, CA) followed by anti-mouse-Alexa 488 secondary antibody (Invitrogen, Carlsbad, CA) and infected foci were visualized by fluorescence microscopy.

### Construction of recombinant IDE and IDE mutants

Construction of recombinant baculovirus expressing HA-tagged human IDE (rIDE) has been reported elsewhere [3] and was used to infect SF9 or H5 insect cells. Three days post-infection the cells were collected, treated with lysis buffer (25 mM Tris-HCl pH 7.4, 5 mM EDTA, 15 mM NaCl, and 0.1% NP40), and the lysate was centrifuged at 2,450 g for 30 min at 4°C. HA-tagged rIDE was purified from the cleared supernatant through an anti-HA conjugated affinity column (Sigma, St. Louis, MO) at 4°C. After extensive washing with PBS, rIDE was eluted with HA peptide (200 µg/ml in PBS). Excess HA peptide was removed using a Centriprep column with a 10 kDa molecular weight cut-off (Millipore, Billerica, MA). For some experiments HA-IDE was further purified through a gel filtration column in 25 mM Tris, pH 8.0, 150 mM NaCl buffer.

Expression and purification of polyhistidine-tagged human wild-type (IDE-w.t.) and mutant (IDE-E111Q) IDE proteins in E. coli were performed as previously described [12] with minor modifications. Briefly, plasmids pProEX-IDEwt and pProEX-IDE-E111Q [12] were propagated in E. coli Rosetta (DE3) cells and protein expression was induced with isopropyl-1-thiogalactopyranoside at a final concentration of 200 µM for 16 hrs. The cells were then lysed in RIPA buffer (10 mM Tris-HCl, pH 8.0, 100 mM NaCl, 1 mM EDTA, 0.1% NP40, 0.5% deoxycholic acid, 0.5% SDS), and polyhistidine-tagged IDE was purified through a Talon metal affinity column (Clontech, Mountain View, CA), eluted with 250 mM imidazole, and dialyzed overnight in PBS at 4°C.

### Purification and biotinylation of gEt protein

Insect SF9 cells were infected with a recombinant baculovirus expressing the extracellular domain of VZV gE with a C-terminal (histidine)_6_ tag [64]. Three days after infection, tissue culture supernatant was harvested and gEt protein was purified through a Talon metal affinity column (Clontech, Mountain View, CA). After extensive washing with 5 mM imidazole diluted in PBS and PBS buffer alone, 0.5 mg/ml of EZ-Link Sulfo-NHS-Biotin in PBS (pH 8.0) (Pierce, Rockford, IL) was added to the resin and incubated for 30 min at 25°C followed by three washes with cold PBS. The biotinylated protein was then eluted with 250 mM imidazole and dialyzed overnight in PBS.

### ELISA assay to detect gE-Fc fusion proteins and rIDE interactions

gE-Fc and control (vaccinia P7.5 and EBV BZLF2) Fc fusion proteins were described previously [3]. Serial dilutions of Fc fusion proteins were coated onto an ELISA plate at 4°C overnight. After washing with PBS containing 0.05% Tween-20, anti-human Fc-horseradish peroxidase was added (Pierce, Rockford, IL). The amount of Fc fusion protein, designated as specific Fc units, was measured spectrophotometrically at OD450 nm after adding substrate-chromogen TMB (Dakocytomation, Carpinteria, CA).

rIDE was coated onto an ELISA plate at 500 ng/well at 4°C overnight. After washing with PBS containing 0.05% Tween-20 and blocking with 5% BSA, Fc fusion proteins were added at the same specific units (see above) at room temperature for 45 min to allow binding, followed by addition of anti-human Fc-horse radish peroxidase and TMB substrate.

### Insulin degradation assay

rIDE was purified from baculovirus-infected cells using an anti-HA affinity column and eluted with HA peptide [3]. IDE from rat liver was purified by multiple chromatographic steps as described previously [13]. Recombinant human IDE containing hexahistidine and FLAG epitopes fused to the amino terminus of IDE was expressed in HEK-293T cells and purified using cobalt affinity chromatography [14]. Protein concentrations were determined using the BCA protein assay kit (Pierce, Rockford, IL). For degradation assays, samples of IDE were incubated with ^125^I-labeled insulin for 15 min at 37°C. IDE preparations were each diluted to attain an equal level of insulin degradation. Samples were resolved by reverse-phase chromatography using a C8 column as described previously [65]. Fractions were collected and the radioactivity was counted. The degree of insulin degradation was defined as the proportion of remaining intact insulin to total recovered radioactivity. The pmoles of insulin degraded in each sample was calculated from the specific activity of the ^125^I-labeled insulin.

### Myristylation assay of gE

MeWo cells were infected with cell-associated VZV ROka or ROka68D32-71 for 24 hr, labeled with ^3^H-9,10 myristic acid (167 µCi/ml) (Perkin-Elmer, Waltham, MA) for 18 hr, and lysed in RIPA buffer. VZV gE was immunoprecipitated with gE monoclonal antibody (Chemicon, Temecula, CA) and protein A-Sepharose beads (Sigma-Aldrich, St. Louis, MO), and separated on a 4–20% SDS-PAGE gel (Invitrogen, Carlsbad, CA). The gel was incubated with ^3^[H]-ENHANCE (Perkin-Elmer, Waltham, MA) according to manufacturer's instructions before autoradiography.

### Immuno-electron microscopy

Human melanoma cells were seeded on Thermanox coverslips (Nalge Nunc International, Rochester, NY) and infected with cell-associated VZV ROka68D32-71 or parental virus (ROka). When similar CPE was noted, cells were fixed with 2% paraformaldehyde (Electron Microscopy Sciences, Hatfield, PA) on ice for 20 min. The coverslips were then incubated with 5% milk, and stained with anti-gE antibody (Millipore, Billerica, MA) and FluoroNanogold conjugated-anti-mouse Fab'-AlexaFluor488 (Nanoprobes, Yaphank, NY) followed by a final fixation with 4% paraformaldehyde, 2.5% glutaraldehyde in 0.1 M sodium cacodylate buffer overnight at 4°C. Samples were washed three times with distilled water followed by 0.01 M sodium citrate and silver enhanced for 4 min with HQ silver reagents (Nanoprobes, Yaphank, NY). Samples were post-fixed with 1.0% osmium tetroxide/0.8% potassium ferrocyanide in 0.1 M sodium cacodylate, dehydrated with a graded ethanol series, and embedded in Spurr's resin. Thin sections were cut with an RMC MT-7000 ultramicrotome (Ventana, Tucson, AZ), and stained with 1% uranyl acetate prior to viewing at 80 kV on a Hitachi H-7500 transmission electron microscope (Hitachi, Tokyo, Japan). Digital images were acquired with a Hammamatsu XR-100 bottom mount digital camera system (Advanced Microscopy Techniques, Danvers, MA) and processed using Adobe Photoshop v. 7 (Adobe Systems Inc, San Jose, CA).

### Real-time PCR to detect VZV internalization

VZV internalization was assayed by modifying a previous protocol used to determine internalization of Kaposi's sarcoma-associated herpesvirus [66]. Cell-free VZV preparations, filtered through a 5.0 µM non-pyrogenic filter (Pall Corporation, Cornwall, UK), were added to MeWo cells seeded in 6-well plates in SPGC buffer on ice for 60 min to allow binding. Cell entry was initiated by raising the incubation temperature to 37°C for the indicated time. Extracellular virus was removed by treating the cells with 300 µg/ml heparin in PBS on ice for 20 min, washing with PBS, inactivating virus with low pH Na-Citrate buffer (40 mM sodium citrate, 10 mM KCl; 135 mM NaCl, pH 3.6) at room temperature for 2 min [67], and washing with PBS. Extracellular VZV viral DNA was removed by incubating the cells with 100 µg/ml proteinase K in HBSS buffer (Invitrogen, Carlsbad, CA) with 1 mM CaCl_2_ at room temperature for 5 min, washing with PBS, and adding 4 units of DNase I per sample at room temperature for 10 min (Roche Applied Science, Indianapolis, IN). Intracellular VZV viral DNA was then extracted together with cellular genomic DNA using a DNeasy blood & tissue kit (Qiagen, Valencia, CA) following the manufacturer's protocol. The copy number of intracellular VZV genomic DNA was determined by real-time PCR using primers and a probe targeting VZV ORF62 as described previously [68]. Serial dilutions of plasmid pCMV62 were used to generate a standard curve, and copy numbers of VZV genomic DNA were normalized using the copy number of human RNaseP DNA amplified from the same samples. The effectiveness of heparin treatment and acid inactivation to remove un-internalized virus, and proteinase K followed by DNase I digestion to remove extracellular viral DNA was verified by quantitative PCR with DNA extracted from the virus-bound cells (virus binding on ice for 60 minutes without shifting to 37°C).

### Limited proteolysis to detect conformational changes in ligand-receptor interactions

Pulse proteolysis [20] of gEt was performed with the following modifications. Biotinylated gEt protein was incubated with rIDE or control proteins at room temperature or 37°C for 30 min followed by adding equal volume of 6 M urea for 18 hr at room temperature. Thermolysin diluted in buffer (2.5 M NaCl, 10 mM CaCl_2_) was added at 0.2 µg/ml at room temperature for 30 sec. The digestion was then terminated by adding 0.5 M EDTA and samples were boiled in SDS-PAGE gel loading buffer with 2.5% 2-mercaptoethanol and separated by electrophoresis. Protein fragments were detected by streptavidin conjugated-horse radish peroxidase.

### Membrane fusion assay

A membrane fusion assay using a β-galactosidase reporter was adapted from Feng et. al. [69]. The pG1N-T7-β-gal plasmid encodes β-galactosidase under the T7 promoter and was a gift from Ed Berger (NIAID, NIH, Bethesda, MD) [70]. Plasmid pAR3126 encodes T7 polymerase driven by an SV40 early promoter [71] and was kindly supplied by William Studier (Brookhaven National Laboratory, Upton, New York). MeWo cells stably expressing T7 polymerase were generated by co-transfecting pAR3126 and pCI-Neo (Promega, Madison, WI) followed by neomycin selection at 1.0 mg/ml. VZV ROka or ROka68Δ32-71 was propagated and titrated to equivalent PFU (plaque forming units) in the MeWo cells expressing T7 polymerase and served as effecter cells. VZV permissive HeLa cells were transfected with pG1N-T7-β-gal for 24 hr and served as target cells. Effecter cells (VZV-infected MeWo cells expressing T7 polymerase) were mixed with target cells (HeLa cells with the β–galactosidase gene driven by the T7 promoter) for 20-24 hrs at 37°C. Cells were then lysed in PBS containing 0.01% NP40, and β- galactosidase expression resulting from fusion of effecter with target cells was measured by incubating the lysate with chlorophenol red-β-D-galactopyranoside (Roche Applied Science, Indianapolis, IN) and performing spectrophotometry at OD570 nm.

## References

[1] Cohen JI, Straus SE, Arvin AM, Knipe DM, Howley PM (2007). Varicella-Zoster Virus Replication, Pathogenesis, and Management.. Fields Virology.

[2] Chen JJ, Zhu Z, Gershon AA, Gershon MD (2004). Mannose 6-phosphate receptor dependence of varicella zoster virus infection in vitro and in the epidermis during varicella and zoster.. Cell.

[3] Li Q, Ali MA, Cohen JI (2006). Insulin degrading enzyme is a cellular receptor mediating varicella-zoster virus infection and cell-to-cell spread.. Cell.

[4] Mallory S, Sommer M, Arvin AM (1997). Mutational analysis of the role of glycoprotein I in varicella-zoster virus replication and its effects on glycoprotein E conformation and trafficking.. J Virol.

[5] Ali MA, Li Q, Fischer ER, Cohen JI (2009). The insulin degrading enzyme binding domain of varicella-zoster virus (VZV) glycoprotein E is important for cell-to-cell spread and VZV infectivity, while a glycoprotein I binding domain is essential for infection.. Virology.

[6] Li Q, Krogmann T, Ali MA, Tang WJ, Cohen JI (2007). The amino terminus of varicella-zoster virus (VZV) glycoprotein E is required for binding to insulin-degrading enzyme, a VZV receptor.. J Virol.

[7] Berarducci B, Ikoma M, Stamatis S, Sommer M, Grose C (2006). Essential functions of the unique N-terminal region of the varicella-zoster virus glycoprotein E ectodomain in viral replication and in the pathogenesis of skin infection.. J Virol.

[8] Berarducci B, Rajamani J, Zerboni L, Che X, Sommer M (2009). Functions of the unique N-terminal region of glycoprotein E in the pathogenesis of varicella-zoster virus infection.. Proc Natl Acad Sci U S A.

[9] Chesneau V, Rosner MR (2000). Functional human insulin-degrading enzyme can be expressed in bacteria.. Protein Expr Purif.

[10] Perlman RK, Rosner MR (1994). Identification of zinc ligands of the insulin-degrading enzyme.. J Biol Chem.

[11] Leissring MA, Farris W, Wu X, Christodoulou DC, Haigis MC (2004). Alternative translation initiation generates a novel isoform of insulin-degrading enzyme targeted to mitochondria.. Biochem J.

[12] Li P, Kuo WL, Yousef M, Rosner MR, Tang WJ (2006). The C-terminal domain of human insulin degrading enzyme is required for dimerization and substrate recognition.. Biochem Biophys Res Commun.

[13] Bennett RG, Duckworth WC, Hamel FG (2000). Degradation of amylin by insulin-degrading enzyme.. J Biol Chem.

[14] Bennett RG, Heimann DG, Hamel FG (2009). Degradation of relaxin family peptides by insulin-degrading enzyme.. Ann N Y Acad Sci.

[15] Duckworth WC, Kitabchi AE (1974). Insulin and glucagon degradation by the same enzyme.. Diabetes.

[16] Farris W, Mansourian S, Chang Y, Lindsley L, Eckman EA (2003). Insulin-degrading enzyme regulates the levels of insulin, amyloid beta-protein, and the beta-amyloid precursor protein intracellular domain in vivo.. Proc Natl Acad Sci U S A.

[17] Dimitrov DS (2004). Virus entry: molecular mechanisms and biomedical applications.. Nat Rev Microbiol.

[18] Marsh M, Helenius A (2006). Virus entry: open sesame.. Cell.

[19] Cole NL, Grose C (2003). Membrane fusion mediated by herpesvirus glycoproteins: the paradigm of varicella-zoster virus.. Rev Med Virol.

[20] Park C, Marqusee S (2006). Quantitative determination of protein stability and ligand binding by pulse proteolysis.. Curr Protoc Protein Sci Chapter.

[21] Delos SE, Godby JA, White JM (2005). Receptor-induced conformational changes in the SU subunit of the avian sarcoma/leukosis virus A envelope protein: implications for fusion activation.. J Virol.

[22] Matsuyama S, Taguchi F (2009). Two-step conformational changes in a coronavirus envelope glycoprotein mediated by receptor binding and proteolysis.. J Virol.

[23] Perlman RK, Gehm BD, Kuo WL, Rosner MR (1993). Functional analysis of conserved residues in the active site of insulin-degrading enzyme.. J Biol Chem.

[24] Harper DR, Kangro HO (1990). Lipoproteins of varicella-zoster virus.. J Gen Virol.

[25] Li Q, Turk SM, Hutt-Fletcher LM (1995). The Epstein-Barr virus (EBV) BZLF2 gene product associates with the gH and gL homologs of EBV and carries an epitope critical to infection of B cells but not of epithelial cells.. J Virol.

[26] Spear PG (2004). Herpes simplex virus: receptors and ligands for cell entry.. Cell Microbiol.

[27] Carpenter JE, Henderson EP, Grose C (2009). Enumeration of an extremely high particle-to-PFU ratio for Varicella-zoster virus.. J Virol.

[28] Maresova L, Pasieka TJ, Homan E, Gerday E, Grose C (2005). Incorporation of three endocytosed varicella-zoster virus glycoproteins, gE, gH, and gB, into the virion envelope.. J Virol.

[29] Sato H, Callanan LD, Pesnicak L, Krogmann T, Cohen JI (2002). Varicella-zoster virus (VZV) ORF17 protein induces RNA cleavage and is critical for replication of VZV at 37 degrees C but not 33 degrees C.. J Virol.

[30] Cohen JI, Sato H, Srinivas S, Lekstrom K (2001). Varicella-zoster virus (VZV) ORF65 virion protein is dispensable for replication in cell culture and is phosphorylated by casein kinase II, but not by the VZV protein kinases.. Virology.

[31] Keller PM, Neff BJ, Ellis RW (1984). Three major glycoprotein genes of varicella-zoster virus whose products have neutralization epitopes.. J Virol.

[32] Dveksler GS, Gagneten SE, Scanga CA, Cardellichio CB, Holmes KV (1996). Expression of the recombinant anchorless N-terminal domain of mouse hepatitis virus (MHV) receptor makes hamster of human cells susceptible to MHV infection.. J Virol.

[33] Clapham PR, McKnight A, Weiss RA (1992). Human immunodeficiency virus type 2 infection and fusion of CD4-negative human cell lines: induction and enhancement by soluble CD4.. J Virol.

[34] Snitkovsky S, Young JA (1998). Cell-specific viral targeting mediated by a soluble retroviral receptor-ligand fusion protein.. Proc Natl Acad Sci U S A.

[35] Damico R, Bates P (2000). Soluble receptor-induced retroviral infection of receptor-deficient cells.. J Virol.

[36] Geraghty RJ, Krummenacher C, Cohen GH, Eisenberg RJ, Spear PG (1998). Entry of alphaherpesviruses mediated by poliovirus receptor-related protein 1 and poliovirus receptor.. Science.

[37] Shukla D, Liu J, Blaiklock P, Shworak NW, Bai X (1999). A novel role for 3-O-sulfated heparan sulfate in herpes simplex virus 1 entry.. Cell.

[38] Tiwari V, O'Donnell C, Copeland RJ, Scarlett T, Liu J (2007). Soluble 3-O-sulfated heparan sulfate can trigger herpes simplex virus type 1 entry into resistant Chinese hamster ovary (CHO-K1) cells.. J Gen Virol.

[39] Lopez M, Cocchi F, Avitabile E, Leclerc A, Adelaide J (2001). Novel, soluble isoform of the herpes simplex virus (HSV) receptor nectin1 (or PRR1-HIgR-HveC) modulates positively and negatively susceptibility to HSV infection.. J Virol.

[40] Kwon H, Bai Q, Baek HJ, Felmet K, Burton EA (2006). Soluble V domain of Nectin-1/HveC enables entry of herpes simplex virus type 1 (HSV-1) into HSV-resistant cells by binding to viral glycoprotein D.. J Virol.

[41] Earp LJ, Delos SE, Park HE, White JM (2005). The many mechanisms of viral membrane fusion proteins.. Curr Top Microbiol Immunol.

[42] Kirschner AN, Sorem J, Longnecker R, Jardetzky TS (2009). Structure of Epstein-Barr virus glycoprotein 42 suggests a mechanism for triggering receptor-activated virus entry.. Structure.

[43] Pertel PE, Fridberg A, Parish ML, Spear PG (2001). Cell fusion induced by herpes simplex virus glycoproteins gB, gD, and gH-gL requires a gD receptor but not necessarily heparan sulfate.. Virology.

[44] Subramanian RP, Geraghty RJ (2007). Herpes simplex virus type 1 mediates fusion through a hemifusion intermediate by sequential activity of glycoproteins D, H, L, and B.. Proc Natl Acad Sci U S A.

[45] Maresova L, Pasieka TJ, Grose C (2001). Varicella-zoster Virus gB and gE coexpression, but not gB or gE alone, leads to abundant fusion and syncytium formation equivalent to those from gH and gL coexpression.. J Virol.

[46] Kaletsky RL, Simmons G, Bates P (2007). Proteolysis of the Ebola virus glycoproteins enhances virus binding and infectivity.. J Virol.

[47] Schornberg K, Matsuyama S, Kabsch K, Delos S, Bouton A (2006). Role of endosomal cathepsins in entry mediated by the Ebola virus glycoprotein.. J Virol.

[48] Pager CT, Craft WW, Patch J, Dutch RE (2006). A mature and fusogenic form of the Nipah virus fusion protein requires proteolytic processing by cathepsin L.. Virology.

[49] Simmons G, Gosalia DN, Rennekamp AJ, Reeves JD, Diamond SL (2005). Inhibitors of cathepsin L prevent severe acute respiratory syndrome coronavirus entry.. Proc Natl Acad Sci U S A.

[50] Kawase M, Shirato K, Matsuyama S, Taguchi F (2009). Protease-mediated entry via the endosome of human coronavirus 229E.. J Virol.

[51] Rojek JM, Pasqual G, Sanchez AB, Nguyen NT, de la Torre JC (2009). Targeting the proteolytic processing of the viral glycoprotein precursor is a promising novel anti-viral strategy against arenaviruses.. J Virol.

[52] Golden JW, Linke J, Schmechel S, Thoemke K, Schiff LA (2002). Addition of exogenous protease facilitates reovirus infection in many restrictive cells.. J Virol.

[53] Townsley AC, Moss B (2007). Two distinct low-pH steps promote entry of vaccinia virus.. J Virol.

[54] Milne RS, Nicola AV, Whitbeck JC, Eisenberg RJ, Cohen GH (2005). Glycoprotein D receptor-dependent, low-pH-independent endocytic entry of herpes simplex virus type 1.. J Virol.

[55] Nicola AV, Straus SE (2004). Cellular and viral requirements for rapid endocytic entry of herpes simplex virus.. J Virol.

[56] Hambleton S, Steinberg SP, Gershon MD, Gershon AA (2007). Cholesterol dependence of varicella-zoster virion entry into target cells.. J Virol.

[57] Mothes W, Boerger AL, Narayan S, Cunningham JM, Young JA (2000). Retroviral entry mediated by receptor priming and low pH triggering of an envelope glycoprotein.. Cell.

[58] Narayan S, Barnard RJ, Young JA (2003). Two retroviral entry pathways distinguished by lipid raft association of the viral receptor and differences in viral infectivity.. J Virol.

[59] Lynch JA, George AM, Eisenhauer PB, Conn K, Gao W (2006). Insulin degrading enzyme is localized predominantly at the cell surface of polarized and unpolarized human cerebrovascular endothelial cell cultures.. J Neurosci Res.

[60] Harpaz R, Ortega-Sanchez IR, Seward JF (2008). Prevention of herpes zoster: recommendations of the Advisory Committee on Immunization Practices (ACIP).. MMWR Recomm Rep.

[61] Santos RA, Hatfield CC, Cole NL, Padilla JA, Moffat JF (2000). Varicella-zoster virus gE escape mutant VZV-MSP exhibits an accelerated cell-to-cell spread phenotype in both infected cell cultures and SCID-hu mice.. Virology.

[62] Pasieka TJ, Maresova L, Shiraki K, Grose C (2004). Regulation of varicella-zoster virus-induced cell-to-cell fusion by the endocytosis-competent glycoproteins gH and gE.. J Virol.

[63] Cohen JI (1998). Infection of cells with varicella-zoster virus down-regulates surface expression of class I major histocompatibility complex antigens.. J Infect Dis.

[64] Kimura H, Straus SE, Williams RK (1997). Varicella-zoster virus glycoproteins E and I expressed in insect cells form a heterodimer that requires the N-terminal domain of glycoprotein I.. Virology.

[65] Hamel FG, Upward JL, Bennett RG (2003). In vitro inhibition of insulin-degrading enzyme by long-chain fatty acids and their coenzyme A thioesters.. Endocrinology.

[66] Veettil MV, Sadagopan S, Sharma-Walia N, Wang FZ, Raghu H (2008). Kaposi's sarcoma-associated herpesvirus forms a multimolecular complex of integrins (alphaVbeta5, alphaVbeta3, and alpha3beta1) and CD98-xCT during infection of human dermal microvascular endothelial cells, and CD98-xCT is essential for the postentry stage of infection.. J Virol.

[67] McClain DS, Fuller AO (1994). Cell-specific kinetics and efficiency of herpes simplex virus type 1 entry are determined by two distinct phases of attachment.. Virology.

[68] Wang K, Lau TY, Morales M, Mont EK, Straus SE (2005). Laser-capture microdissection: refining estimates of the quantity and distribution of latent herpes simplex virus 1 and varicella-zoster virus DNA in human trigeminal Ganglia at the single-cell level.. J Virol.

[69] Feng Y, Broder CC, Kennedy PE, Berger EA (1996). HIV-1 entry cofactor: functional cDNA cloning of a seven-transmembrane, G protein-coupled receptor.. Science.

[70] Nussbaum O, Broder CC, Berger EA (1994). Fusogenic mechanisms of enveloped-virus glycoproteins analyzed by a novel recombinant vaccinia virus-based assay quantitating cell fusion-dependent reporter gene activation.. J Virol.

[71] Kalderon D, Roberts BL, Richardson WD, Smith AE (1984). A short amino acid sequence able to specify nuclear location.. Cell.

